# Dendritic cells release exosomes together with phagocytosed pathogen; potential implications for the role of exosomes in antigen presentation

**DOI:** 10.1080/20013078.2020.1798606

**Published:** 2020-07-26

**Authors:** Marthe F. S. Lindenbergh, Richard Wubbolts, Ellen G. F. Borg, Esther M. van ’T Veld, Marianne Boes, W. Stoorvogel

**Affiliations:** a Department Biomolecular Health Sciences, Faculty of Veterinary Medicine, Utrecht University, Utrecht, The Netherlands; b Department of Pediatrics and Laboratory of Translational Immunology, University Medical Center Utrecht, University Utrecht, Utrecht, The Netherlands

**Keywords:** Dendritic cells, antigen presentation, exosomes, phagocytosis

## Abstract

Dendritic cells (DC) have the unique capacity to activate naïve T cells by presenting T cell receptor specific peptides from exogenously acquired antigens bound to Major Histocompatibility Complex (MHC) molecules. MHC molecules are displayed on the DC plasma membrane as well as on extracellular vesicles (EV) that are released by DC, and both have antigen-presenting capacities. However, the physiological role of antigen presentation by EV is still unclear. We here demonstrate that the release of small EV by activated DC is strongly stimulated by phagocytic events. We show that, concomitant with the enhanced release of EV, a significant proportion of phagocytosed bacteria was expulsed back into the medium. High-resolution fluorescence microscopic images revealed that bacteria in phagosomes were surrounded by EV marker-proteins. Moreover, expulsed bacteria were often found associated with clustered HLA II and CD63. Together, these observations suggest that exosomes may be formed by the inward budding into phagosomes, whereupon they are secreted together with the phagosomal content. These findings may have important implications for selective loading of peptides derived from phagocytosed pathogens onto exosome associated HLA molecules, and have important implications for vaccine design.

## Introduction

Dendritic cells (DC) are professional antigen presenting cells that use their innate immune functions to drive adaptive immune responses [1]. Once activated, for example upon recognition of components from invading pathogens by Toll-like receptors (TLRs), DC have unique abilities to excise peptides from proteins of exogenous origin that are acquired by endocytic processes, load these peptides onto major histocompatibility complex (MHC) molecules, and present the resulting complexes to activate cognate naïve T cells. Like most cell types, DC release heterogeneous populations of extracellular vesicles (EV). EV include microvesicles (MV) that pinch off from the plasma membrane, and exosomes that are secreted by multivesicular endosomes [2–4]. EV from activated DC carry MHC-peptide complexes that can activate T cells by their own right or after being recruited by and in association with bystander DC [5–7]. DC-derived EV are heterogeneous in size and molecular composition, and in their capacities to stimulate T cells [8–10]. Their functionality is also dependent on the status of maturation of the producing DC [10–12]. EV isolated from cultured DC have been tested for their potential use as vaccine against cancer and pathogens [2,6,13–15]. Yet, the precise role that DC-derived EV play *in vivo* remains unclear. Previously, we reported that interactions of DC with activated T-cells stimulated the release of antigen presenting EV, suggesting a role EV in dissemination and reinforcement of antigen presenting potential within lymphoid tissues [16,17]. Another hint for antigen presenting functions of EV came from observations that both macrophages and epithelial cells can release phagocytosed pathogens through non-lytic expulsion, involving direct fusion of the phagosome with the plasma membrane [18–24]. Interestingly, it has been described that exosomes may be co- released by that process [25]. In our current study we demonstrate for the first time that in response to being challenged by *Escherichia coli*, DC expulse a significant proportion of previously phagocytosed *E. coli*. Moreover, we show that this process coincides with the release of exosomes. Potentially, these observations have implications for preferential loading of pathogen-derived antigens onto exosome associated MHC molecules, and the efficacy of such generated exosomes in antigen presentation.

## Materials and methods

### Cell culture and processing

Blood from healthy volunteers was obtained following institutional ethical approval (METC protocol number 07–125/C). The experiments abide by the Declaration of Helsinki principles for human research ethics. Peripheral blood mononuclear cells (PBMCs) were isolated from lithium heparinized blood samples using Ficoll isopaque density gradient centrifugation (GE Healthcare). CD14+ monocytes were isolated by positive selection using CD14+ MicroBeads (Miltenyi Biotec). The purity of CD14+ sorted cells was determined using flow cytometry after staining for CD14 and CD3. Only cultures containing ≥90% CD14+ cells prior to differentiation towards monocyte derived DC (moDC) were used for further experimentation. For differentiation into moDC [26], CD14+ cells were cultured in 6-wells plates (Nunclon, Thermo Scientific) at a concentration of 1–1.5 × 10^6^/ml in 2–3ml per well at 37°C and 5% CO_2_ in RPMI 1640 GlutaMAX (Gibco), 1% Penicillin/Streptomycin (Gibco) and 20% heat inactivated, 0,2 µM filtered FCS (Biowest), supplemented with 450U/ml rhGM-CSF (Immunotools) and 300U/ml IL-4 (Immunotools) for a total of five days. EV-depleted FCS was prepared after fivefold dilution in RPMI by centrifugation for 18 hours at 100,000 × *g* in polyallomer tubes (Beckman Coulter) using a swing-out rotor (SW-28, Beckman Coulter). Cytokines were replenished after three days. Prior to experiments, the immature moDC, which were in suspension, were harvested by subtle resuspension in culture medium, and washed once with PBS by centrifugation for 10 min at 330 × *g*. After washing, the immature moDC were resuspended in culture medium containing EV depleted FCS for further culture/experimentation. When indicated, moDC were cultured for 16 hours in the presence or absence of 100 ng/ml ultrapure LPS (from *E. coli* strain O111:B4, Invivogen) and/or heat inactivated *E. coli* (see below). For these experiments, cells were seeded in 6 wells plates in 2ml/well at a density of 100,000 cells/ml.

### *E. coli* preparation

*E. coli* strain DH5α was cultured in LB medium (MP Biomedicals) to a OD600 of 0.5 and then heat-killed by incubating for 30 min at 75°C. Heat killed bacteria were pelleted by centrifugation for 5 min at 13,300 × *g*, resuspended in 0.2M bicarbonate buffer at pH 8.3 using a 23G syringe, and washed in the same buffer using the same procedure. Washed bacteria were labelled either with Cy3B-NHS (200µg/ml, Thermo Fisher), or with a combination of AlexaFluor405-NHS (200µg/ml Thermo Fisher) and EZ-Link NHS-LC-Biotin (88µg/ml Thermo Fisher) for 45 min at 4°C under constant rotation. In case of double labelling, EZ-Link NHS-LC-Biotin was added 10 min after the NHS-AlexaFluor405-NHS ester. After labelling, bacteria were washed three times by centrifugation for 5 min at 13,300 × *g* and resuspension in PBS (phosphate buffered saline, Gibco) containing 100 mM glycine (Sigma). Glycine was added to ensure quenching of non-reacted NHS-groups. Finally, the bacteria were washed three times in PBS prior to storage at 4°C. Bacteria were quantified using a haemocytometer.

### Flow cytometry

After incubation, detached moDC were harvested in excess ice cold PBS. Adherent cells were gently dissociated from the plastic by pipetting in ice cold PBS and pooled with the already detached cells. Viability was probed using 7-Aminoactinomycin D (7-AAD) (1:25, BD) according to manufacturer instructions. All subsequent immune-labelling steps were performed on unfixed cells at 4°C. Prior to incubation with antibodies, cells were blocked in 0.5% NMS (Fitzgerald 88R-M002) diluted in flow cytometry buffer (PBS with 0.5% BSA and 0.02% NaN3). HLA-II was detected with PE-Cy7-labelled mouse-anti human HLA-DR (clone L243; 1:200; BioLegend), CD86 with PE-labelled mouse anti-human CD86 (clone 2331/FUN-1, 1:50, BD), CD11c with fluorescein-5-isothiaocyanate (FITC)-labelled mouse anti-human CD11c (clone BU15, 1:50, Invitrogen), CD14 with FITC-labelled mouse anti-human CD14 (clone Tük 4, 1:100, Miltenyi), and CD3 with pacific blue (PB)-labelled mouse anti-human CD3 (clone UCHT-1, 1:50, Beckman Coulter). PE-labelled murine IgG1,k (clone MOPC-21; BD) and PE-Cy7-labelled murine IgG2a,k (clone MOPC-173; BioLegend) were used as isotype controls. Flow cytometry was performed on a FacsCanto II (BD) flow cytometer and the data were analysed with FlowJo 10.0 software (Treestar).

### Flow cytometry-based analysis of expulsed *E. coli*

MoDC were incubated for 3 hours with *E. coli* that were both biotinylated-, and labelled with AF405 (25 *E. coli* per moDC). The moDC were then resuspended in ice cold PBS, and collected by centrifugation at 240 × *g* for 4 min at 4°C in a tabletop centrifuge. The majority of non-phagocytosed *E. coli* remained in the supernatant by this procedure. The pelleted moDC were resuspended and washed in PBS by centrifugation at 240 × *g*, and probed for 10 min with Streptavidin-labelled allophycocyanin (APC) (1:50, eBiosciences) on ice to label any remaining extracellular non-phagocytosed biotin and AF405 double labelled *E. coli*. After washing away excess streptavidin-APC, the moDC were re-incubated at cell culture conditions for another 3 hours to allow expulsion of phagocytosed APC-negative *E. coli*. Hereafter the samples were centrifuged for 4 min at 240 × *g* to remove moDC. Expulsed *E. coli* was collected from the supernatant by centrifugation at 13,300 × *g* for 8 min and labelled with streptavidin-phycoerythrin (PE) (1:50, eBiosciences) for 10 min on ice. After labelling, *E. coli* was washed once in cold medium and once in PBS, and resuspended in flow cytometry buffer (PBS with 0.5% BSA and 0.02% NaN_3_) for flow cytometric analysis, as described above.

### EV isolation and Western blotting analysis

EV were collected from cell culture media by differential (ultra)centrifugation at 4°C, as reported earlier [27]. MoDC were removed in two subsequent centrifugation steps of 10 min at 200 × *g*. Next, supernatants were centrifuged sequentially two times for 10 min at 500 × *g*, once for 30 min at 10,000 × *g*, and finally for 65 min at 100,000 × *g*. The last two centrifugation steps were performed in polyallomer tubes (Beckman Coulter) using a swing-out rotor (SW-40, Beckman Coulter). The 10,000 × *g* and 100,000 × *g* pellets, predominantly containing large and small EV respectively, were lysed in non-reducing SDS-PAGE sample buffer and incubated for 5 min at 100°C. After separation by 10% SDS-PAGE, proteins were transferred to 0.45 µm polyvinylidene difluoride membranes (Merck Millipore). The blots were blocked in PBS containing 0.2% cold water fish skin gelatin (Sigma) and 0.1% Tween-20. Immuno-labelling was performed in the same buffer using mouse anti-human CD9 (clone HI9a; 1:2000; Biolegend), mouse anti-human CD63 (clone TS63; 1:2000; Abcam), mouse anti-human CD81 (clone B-11; 1:400; Santa Cruz), mouse anti-human HLA-B,C (clone HC-10; 1:400; kindly provided by E.J.H.J. Wiertz), or mouse anti-human HLA II (clone CR3/43; 1:10,000; Dako). Primary antibodies were labelled with HRP-conjugated goat anti-mouse IgG and IgM (1:10,000; Jackson). HRP activity was monitored using ECL (SuperSignal West Dura Extended Duration Substrate, Thermo Scientific) and detected with a ChemiDoc MP Imaging System (BioRad). Relative signal strengths were determined using Image Lab V5.1 (BioRad).

### Structured illumination microscopy (SIM)

MoDC were cultured for approximately 16 hours either in presence or absence of Cy3B-labelled *E. coli* (10 *E. coli*/moDC) in 6-wells culture plates, and subsequently harvested in ice cold PBS. The harvested moDC were pelleted by centrifugation for 10 min at 330 × *g*, and resuspended to 0.5×10^6^ cell/ml in cell culture medium. Next, 200 µl samples of the cell suspension were placed onto 12 mm glass coverslips (World Precision Instruments) using a custom made adaptor (3D design available upon request) fitted onto cuvettes (Shandon-Elliott) that were pre-wetted with 50 µL 2% bovine serum albumin in PBS, and centrifuged for 3 min at 800 rpm and room temperature using a cytocentrifuge (Shandon-Elliott). Subsequently, the coverslips were fixed for 30 min in 4% paraformaldehyde in 0,1 M phosphate buffer, pH 7.4. Fixed cells were washed with PBS and permeabilized for 5 min in PBS containing 0,1% Saponin (Sigma), 2% bovine serum albumin (BSA), and quenched for 20 min in 20mM NH_4_Cl. Subsequent washing and labelling was performed in PBS containing 2% BSA and 0.1% saponin. Cells were labelled with mouse anti human HLA II (CR3/43; 0.4µg/ml, Dako) or mouse anti human CD63 (H5C6, 1µg/ml, BD) for 45 min at room temperature, washed three times and incubated with goat anti-mouse AlexaFluor488 (1µg/ml, Invitrogen). Labelled cells were washed twice in labelling buffer, twice with PBS, and finally in deionized water. Coverslips were mounted onto object glasses in Prolong Diamond embedding media (Thermo Fisher) and left to solidify overnight. Images were acquired using a DeltaVision OMX V4 Blaze imaging system in SIM imaging modus with a 60× objective (Olympus U-PLAN APO, NA 1.42) and oil with a refractive index of 1.516, with the factory-supplied BGR filter tray and 125 nm step size in the Z-axis. Acquisition was executed by the OMX Deltavision software package and images were reconstructed using SoftWoRx (Cytiva) with OTFs generated by GE Healthcare service engineers (raw data, alignment parameters available on request). Movies and maximum intensity projections were prepared in Imaris 8.1 (Andor/Bitplane) with linear intensity adjustments for labelled channels and a gamma correction of 1.4 was applied for *E. coli* channels to highlight bacteria. Cropped regions of reconstructed and channel aligned SIM images of bacteria were generated using FIJI, using its scripting tools to adjust linearly for the different labels (Alx488 1000/15000); Cy3B 7500/60000).

### Live confocal microscopy-based expulsion analysis

MoDC were labelled with cell trace yellow (CTY) (1µM, Thermo Fisher) in PBS at room temperature for 5 min, followed by addition of cold culture medium 1:1 and a further 5 min incubation on ice. Subsequently, cells were pelleted by spinning 10 min at 330 × *g* and resuspended in warm medium. Next, the moDC were seeded in Eppendorf tubes and incubated with biotinylated, AlexaFluor405-labelled *E. coli* (10/moDC) at regular culture conditions. After three hours, moDC were pelleted by centrifugation for 4 at 240 × *g,* washed two times with cold PBS, and stained with AlexaFluor647-labelled streptavidin (1:100, Invitrogen) for 10 min on ice. Subsequently, cells were washed twice with cold medium, resuspended in cold medium and seeded in a Fluorodish (35–100, World Precision Instruments). Fluorodishes were kept on ice until the start of imaging. Imaging was performed on a NIKON A1R confocal microscope with a 40× Plan Apo objective (NA 1.3) while maintaining cell culture conditions (37°C, 5% CO_2_) in a tabletop culture control unit (TOKAI Hit). Confocal scanning was performed using the resonance mode with a pinhole set 4× the airy disk and a pixel size of 0.62 µm. Diode laser and filter settings were used to detect AlexaFluor405, CTY, and AlexaFluor647 sequentially using bidirectional line switching. Differential interference contrast (DIC) images were collected along with the CTY channel. Overviews of the cultures were generated every 2 min by automated scanning of 4×4 image fields in 5 positions along the Z-axis at 1.5µm steps. Imaging data were acquired over a period of up to 11 hours and processed in NIS elements 5.02 (Nikon Microsystems). Images were pre-processed by subtracting the average intensity in the Z-stack before maximal internal projection. Objects were isolated using the spot isolation procedure in NIS elements with background subtraction and median filtering. Alexa647 binary isolates were dilated by 1 pixel to compensate spectral and motion shifts due to sequential recording. Isolated objects were classified based on co-incidence of the channels. Expulsed *E. coli* objects were defined as Alexa405-positive, Alexa647-negative, CTY-negative events, counted over time, normalized to number of counted moDC, and related relative to the average total Alexa 405-positive, Alexa647-negative events (both CTY-positive and CTY-negative) that were recorded in the first hour of imaging.

### Statistical analysis

Significance of difference was determined using Wilcoxon’s signed rank test with GraphPadPrism 7 software. *P* values ≤0.05 were considered statistically significant.

## Results

### MoDC phagocytose E. coli and become activated in the process

**Figure 1.**
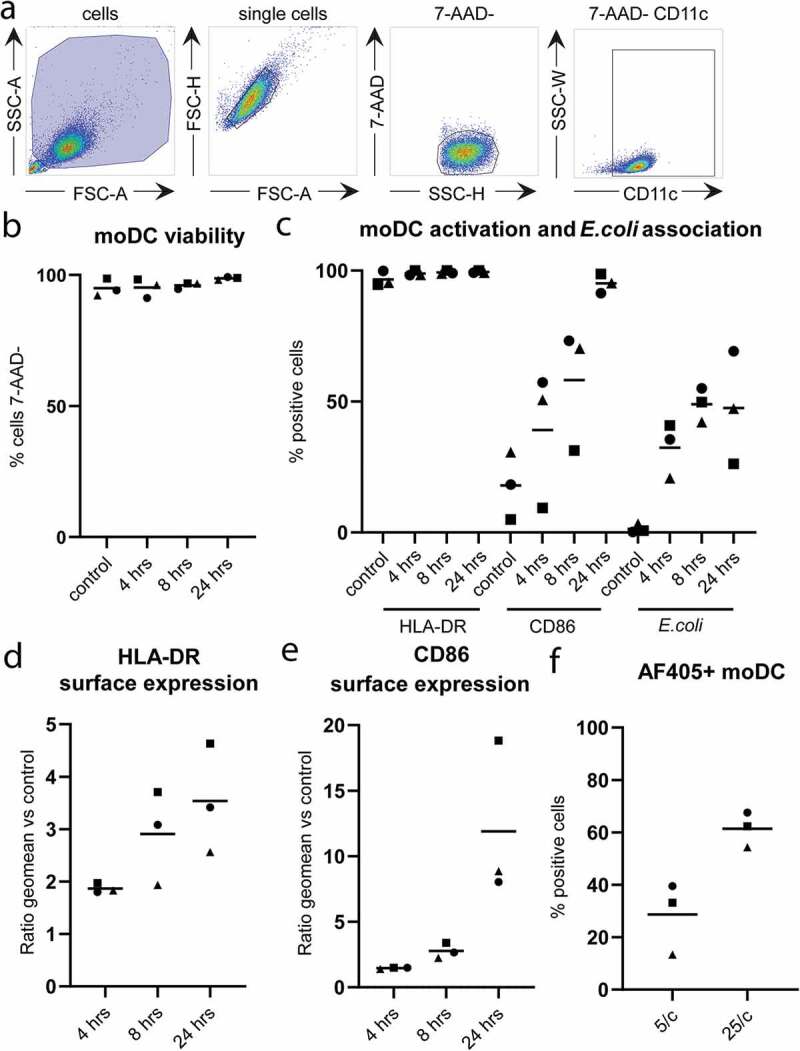
Phagocytosis of *E. coli* and activation of moDC.

To study long-term effects of phagocytosis on the release of EV by moDC, we used fluorescently (AF405) labelled *E. coli* that were heat killed to prevent overgrowth of moDC. CD11c-expressing moDC were detected by flow cytometry (Figure 1(a)). The viability of the moDC, as determined by 7-AAD-exclusion, remained unaffected by 24 hours incubation in the presence of 25 bacteria per moDC (Figure 1(b)). HLA-DR and CD86 were increasingly expressed over time, and nearly all moDC had matured after 24 hours (Figure 1(c-e), supplementary figures 1 and 2). Approximately 50% of the moDC contained a measurable signal for phagocytosed *E. coli* after 8 hours incubation with 25 bacteria per moDC (Figure 1(c,f), supplementary figure 2). Of note, this percentage was significantly lower when moDC were incubated with fewer *E. coli* (Figure 1(f)). The amount of 25 *E. coli* per moDC provided a maximal activation signal, as determined by the observation that the additional presence of LPS did not further enhance surface expression of moDC maturation markers (supplementary figure 1).

(a) Flow cytometric gating strategy for moDC. Cells were first selected on forward and side scatter properties, followed by selection of single cells (FSC-H vs FSC-A). Subsequently, viable single cells were identified by exclusion of 7-AAD. Finally, moDC were selected based on cell surface expression of CD11c. (b-e) moDC were incubated in the absence (control) or presence (4, 8 or 24hours) of AF405 labelled *E. coli* (10 bacteria per moDC), harvested, stained with antibodies, and analysed by flow cytometry as in A. *n*=3 with each experiment indicated by separate symbols and mean values indicated with horizontal lines. (b) Viability of moDC was unaffected by incubation with *E. coli*. (c) All moDC expressed HLA-DR on their surface, irrespective of activation by *E. coli*. The percentage of activated moDC, as determined by expression of CD86, steadily increased to 95% during 24 hour of incubation with *E. coli*. The percentage of moDC that contained *E. coli* increased to 50% during the first 8 hour of incubation, and did not increase further up to 24 hours. Representative flow cytometry plots acquired after 24-hour incubation are shown in supplementary figure 2. (d and e) Geometric mean of HLA-DR and CD86 cell surface expression after incubation with *E. coli* expressed as fold increase relative to control. (f) Percentage of *E. coli* labelled moDC after 24 incubation with either 5 or 25 *E. coli* per moDC. *n*=3, with each experiment indicated by separate symbols and mean values indicated by horizontal lines.

**Figure 2.**
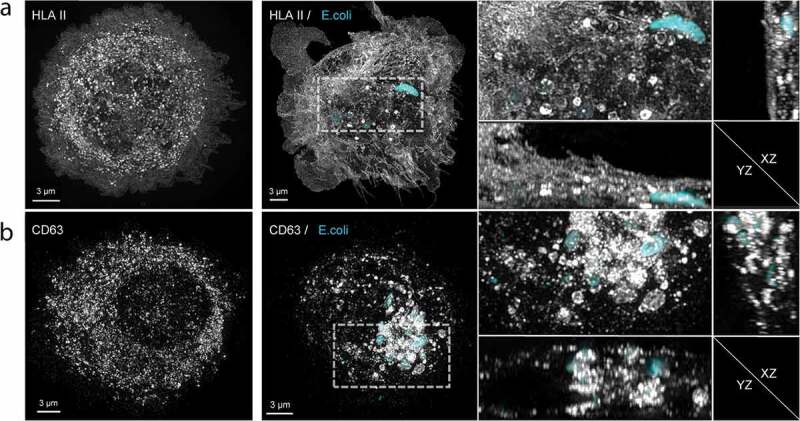
Subcellular distributions of HLA II and CD63 relative to phagocytosed *E. coli.*

**Figure 3.**
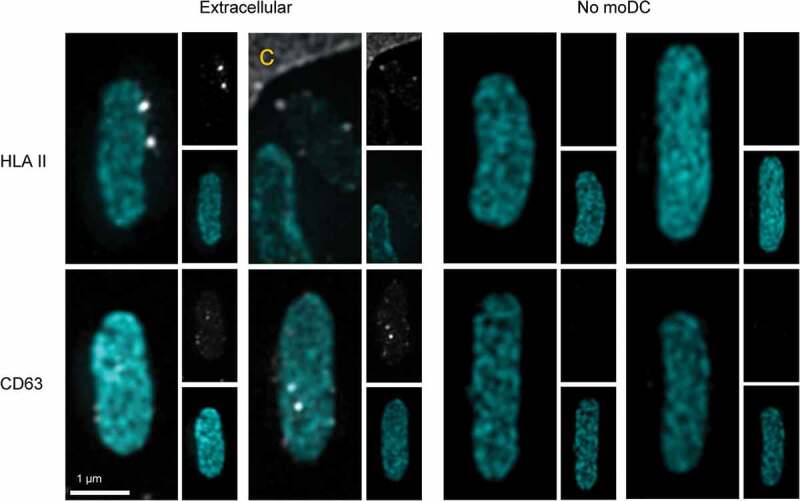
EV markers on expulsed *E. coli.*

Phagocytosis of Cy3B-labelled *E. coli* by moDC was confirmed using structured illumination microscopy (SIM) 3D imaging, wherein moDC were stained for HLA II or CD63 (Figure 2 and supplementary movies 1 and 2). In immature, unstimulated moDC (Figure 2(a) left panel), HLA II (grey) was mostly intracellular. As expected, moDC were activated by 16-hour incubation with *E. coli*, resulting in transfer of HLA II to the plasma membrane. Importantly, phagocytosed *E. coli* (cyan) was often found surrounded by a ring of small HLA II labelled puncta (Figure 2(a), middle and right panels, and supplementary movie 1 for 3D rotation). Similar to HLA II, also CD63 (grey) was found throughout the cell in immature moDC (Figure 2(b) left panel). After phagocytosis of *E. coli*, CD63 redistributed to the phagosome containing areas of the cell, some of which distributed into puncta at the *E. coli* containing phagosomes (Figure 2(b) middle and right panels, and supplementary movie 2 for 3D rotation). CD63 is known to be highly enriched in endosomes as well as in exosomes [28] and thought to be instrumental for cargo selection into exosomes [28,29], triggering the question whether exosomes could be expulsed along with phagocytosed bacteria. Consistent with this idea, some extracellular *E. coli* that had the opportunity to be phagocytosed and expulsed by moDC were decorated with discrete HLA II and CD63 labelled puncta (Figure 3).

Representative flattened maximum intensity 3D SIM images from one experiment out of three independent experiments. (a) The subcellular distributions of HLA II (grey) in an immature moDC (left panel) and in an activated moDC after 16-hour incubation in the presence of Cy3B-labelled *E. coli* (cyan) (middle panel). Note the redistribution of HLA II from endosomal/lysosomal compartments to the plasma membrane in response to activation. The indicated segment is enlarged in the right panel, together with YZ and XZ sections of the same segment. Note the punctate HLA II staining surrounding *E. coli*-containing phagosomes. For 3D rotation see supplementary movie 1. (b) The subcellular distributions of the exosomal marker CD63 (grey) in an immature moDC (left panel) and in an activated moDC (middle panel) upon uptake of *E. coli* (cyan). The indicated segment is enlarged in the right panel, together with YZ and XZ sections of the same segment. Note the recruitment of CD63 towards the phagosome containing area of the moDC, and the punctate staining pattern surrounding the many *E. coli*-containing phagosomes. For 3D rotation see supplementary movie 2.

MoDC were cultured for 16 hour in presence of Cy3B-labelled *E. coli*. Extracellular *E. coli* (cyan) that were captured together with moDC were often decorated with HLA II (grey) and CD63 (grey) puncta (left panels), indicating associated EV. Merged pictures show three and two representative examples for HLA II and CD63 respectively. Single channel pictures are displayed in half size prints for comparison. The edge of one MoDC is indicated with c. Examples of control *E. coli* that had not been incubated with moDC were imaged with identical settings, and the absence of HLA II and CD63 labelling on these EV lacking bacteria demonstrates specificity of the labelling procedure (right panels, indicated with no MoDC).

### Phagocytosis induced release of EV

**Figure 4.**
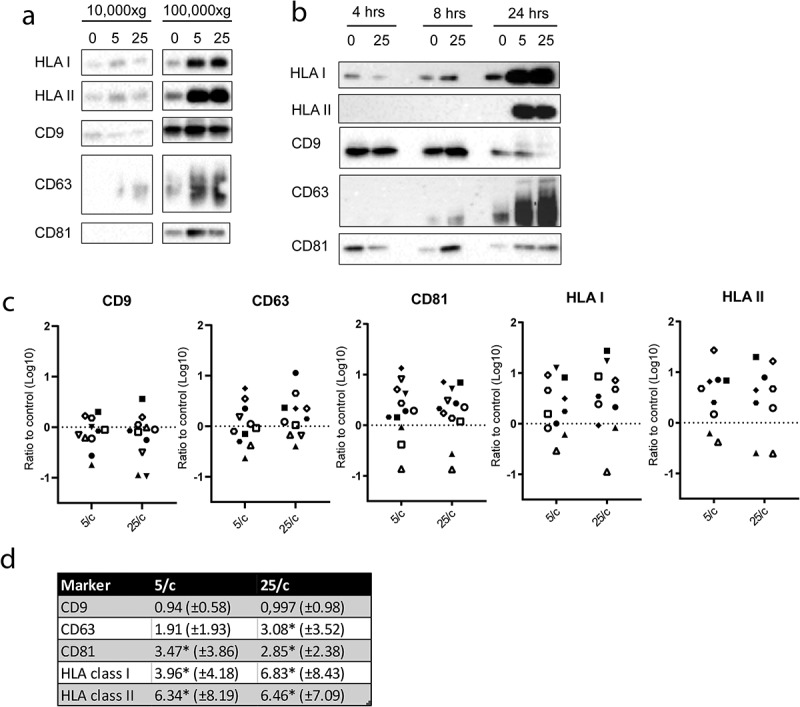
*E. coli* -induced EV release by moDC.

To test whether phagocytosis of *E. coli* influenced the release of free EV, moDC were activated by LPS, either in the absence or presence of 5 or 25 heat inactivated *E. coli* per moDC. Activation of moDC by LPS alone may already affect the release of EV, although this idea is still controversial since only minor stimulatory as well as minor inhibitory effects of LPS on EV release by DC have been reported [30,31]. Therefore, to eliminate any potential bias introduced by moDC activation on EV release, LPS only was taken as control condition. EV were collected from the culture media by differential centrifugation. After removal of cells and cell debris, large EV were pelleted together with any remaining free *E*. coli, when present, by centrifugation at 10,000 × *g*. The supernatant was then re-centrifuged at 100,000 × *g* to collect small EV, including exosomes [12,27]. The 10,000 × *g* and 100,000 × *g* pellets were analysed by immunoblotting for the presence of EV-associated proteins. The antigen presenting molecules HLA-I and HLA-II, and the tetraspanins CD9, CD63, and CD81, were more abundantly present in the 100,000 × *g* compared to 10,000 × *g* pellets (Figure 4(a)). This observation is consistent with predominant association of these markers with small EV [12]. Interestingly, as compared LPS alone, the additional presence of *E. coli* stimulated the release of HLA-I, HLA-II, CD63 and CD81, but not CD9, in association with small EV. These observations indicate that the release of small EV is triggered by phagocytosis of bacteria independently of moDC activation. To determine the kinetics of secretion, moDC were stimulated for 4, 8 or 24 hour with LPS only or in the additional presence of 5 or 25 *E. coli* per moDC. HLA I, HLA II, and CD63 were most abundantly present in the 100,000 × *g* pellet after 24 hours incubation with *E. coli*, whereas the amount of CD9 did not increase over time (Figure 4(b-d)). These data were collected from 11 independent experiments, in which cells from different donors were used. The high variability between these experiments may be due to donor differences. Additionally, it should be noted that, although only batches of moDC with >90% viability were selected for these experiments (as determined by 7-AAD exclusion, see Figure 1(b)), the release of EV from a minor population of dying cells can result in a dramatic increase in background signal for EV release. Indeed, we observed that signals for EV markers increased, most prominently in 10,000 × *g* pellets but also in 100,000 × g pellets, when moDC preparations with viability scores < 90% were used (data not shown). The majority of EV released by lesser quality moDC may represent apoptotic vesicles, and the high background signals imposed by such apoptotic bodies masked contributions of EV that were released by healthy cells in response to *E. coli* phagocytosis. It is likely that even in our moDC preparations that were selected for high viability scores, slight variation in the quality of the cell cultures contributed to variation between the 11 replicates. Nonetheless, we found a dose dependent (5 versus 25 *E. coli* per moDC) and significant increase for CD63, CD81, HLA I, and HLA II in 100,000 × *g* pellets in response to *E. coli* + LPS, as compared to LPS alone (Figure 4(d)). In contrast, the release of CD9 remained unaffected. This is consistent with the notion that CD9 may be predominantly associated with plasma membrane-derived MV rather than with exosomes [12].

(a) Representative immunoblots from 11 independent experiments detecting HLA-I, HLA-II, CD9, CD63 and CD81 in EV isolated from the culture media from moDC that were incubated for 24hours in the presence of LPS only (0) or presence of both LPS and 5 or 25 *E. coli* per moDC. Samples of the sequentially harvested 10,000 × g and 100,000 × g pellets were loaded and detected on the same blot (exemplified in supplementary figure 3). (b) Representative immunoblot of EV markers in 100,000 × g pellets from culture media from moDC that were incubated for 4, 8, or 24hours either in the presence of LPS only (0) or in the presence of both LPS and 5 or 25 *E. coli* per moDC. (c) Quantification of signals as in (b) from 11 independent experiments of appropriately exposed immunoblots of EV pelleted at 100,000 × g from culture media of 24 hour treated moDC. Each symbol corresponds to 1 individual donor. Signal strength induced by 5 or 25 *E. coli* per moDC is plotted relative to the signal in the absence of *E. coli* within the same experiment, and fold increase is expressed on a log10 scale. (d) Statistical analysis of fold increase of signals as determined in c (mean ± SD; * indicates significant increase with p≤0.05).

### Expulsion of phagocytosed E. coli by moDC

**Figure 5.**
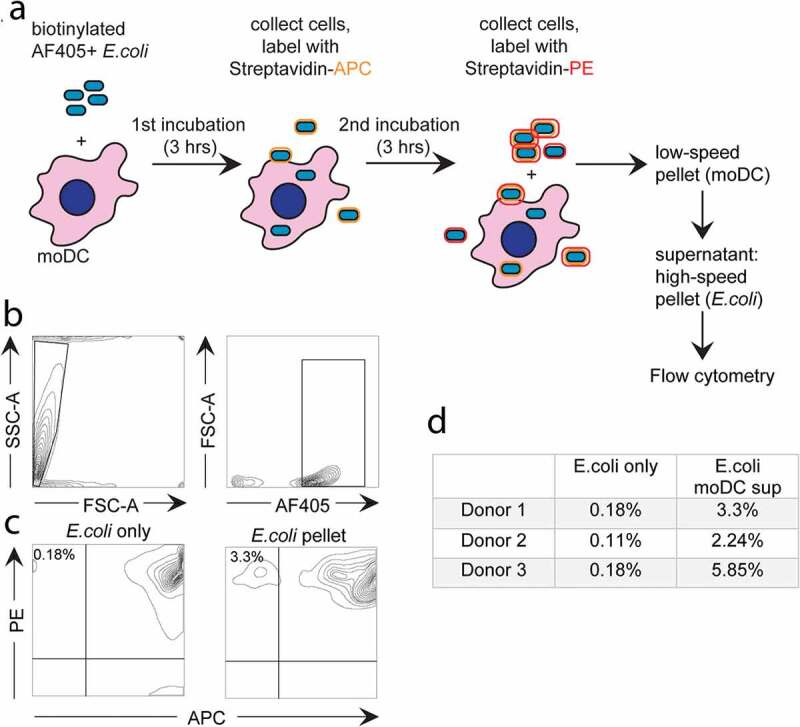
Flow cytometry-based analysis of *E. coli* expulsion by moDC.

When exosomes can indeed be formed as ILV in phagosomes, their release by secreting phagosomes would imply that phagocytosed *E. coli* can be secreted back into the extracellular environment. To investigate this possibility, we first used a flow cytometry-based approach (schematic representation in Figure 5(a)). First, moDC were incubated for 3 hours with *E. coli* that were both labelled with AF405 and biotinylated. The moDC were then separated from the majority of non-phagocytosed extracellular *E. coli* by centrifugation. Those bacteria that were not phagocytosed but co-pelleted with the moDC, either in association with the moDC plasma membrane or as free bacteria, were labelled with streptavidin-APC, leaving truly phagocytosed *E. coli* unlabelled by the streptavidin conjugate. The moDC were then chased for 3 hours at 37°C allowing expulsion of phagocytosed, APC-negative, *E. coli*. After cooling, expulsed *E. coli* were separated from their originating moDC by differential centrifugation, stained with streptavidin-PE, and identified by flow cytometry as AF405^+^, APC^−^, PE^+^ gated events. The AF405^+^ gating strategy is illustrated for a control *E. coli* sample that was labelled in the absence of moDC, demonstrating the efficacy of labelling (Figure 5(b,c), left panels). With this strategy, we detected a small but significant population of *E. coli* that had been expulsed after being phagocytosed by moDC (Figure 5(c) right panel, and Figure 5(d)). These values reflect the amount of recycled *E. coli* relative to the amount of *E. coli* that was not phagocytosed and failed to be removed after pulse loading the moDC. Therefore, although this approach demonstrated that expulsion of phagocytosed bacteria does occur, it did not enable us to quantify the relative amount of phagocytosed *E. coli* that was expulsed.

(a) Schematic presentation of the experimental design. MoDC were incubated for 3hours with *E. coli* that was labelled with both biotin and AF405 (blue). Subsequently, all moDC and some *E. coli* were collected by centrifugation for 8minutes at 260 × g, leaving the majority of non-phagocytosed *E. coli* in the supernatant. Pelleted cells were incubated with streptavidin-APC (orange), labelling those biotinylated *E. coli* that were co-pelleted with the moDC but not phagocytosed. Subsequently, moDC were incubated for another 3hours at 37°C, cooled to 4°C, and stained with streptavidin-PE (red). moDC together with some *E. coli* were removed by centrifugation at 260 × g, and *E. coli* remaining in the moDC supernatant were pelleted by a subsequent centrifugation step at 13,300 × g. Expulsed *E. coli* was identified by flow cytometry as AF405+, APC-, PE+ events. (b) Gating strategy for *E. coli: E. coli*-sized particles were gated based on scatter and AF405 signal. (c) Flow cytometry plots showing labelling efficiency in a control (“*E. coli* only”, left panel) and expulsed *E. coli* (APC-, PE+, “E. *coli pellet*”, right panel). (d) Amount of expulsed *E. coli* as percentage of total extracellular *E. coli*, according to three independent experiments as in c.

**Figure 6.**
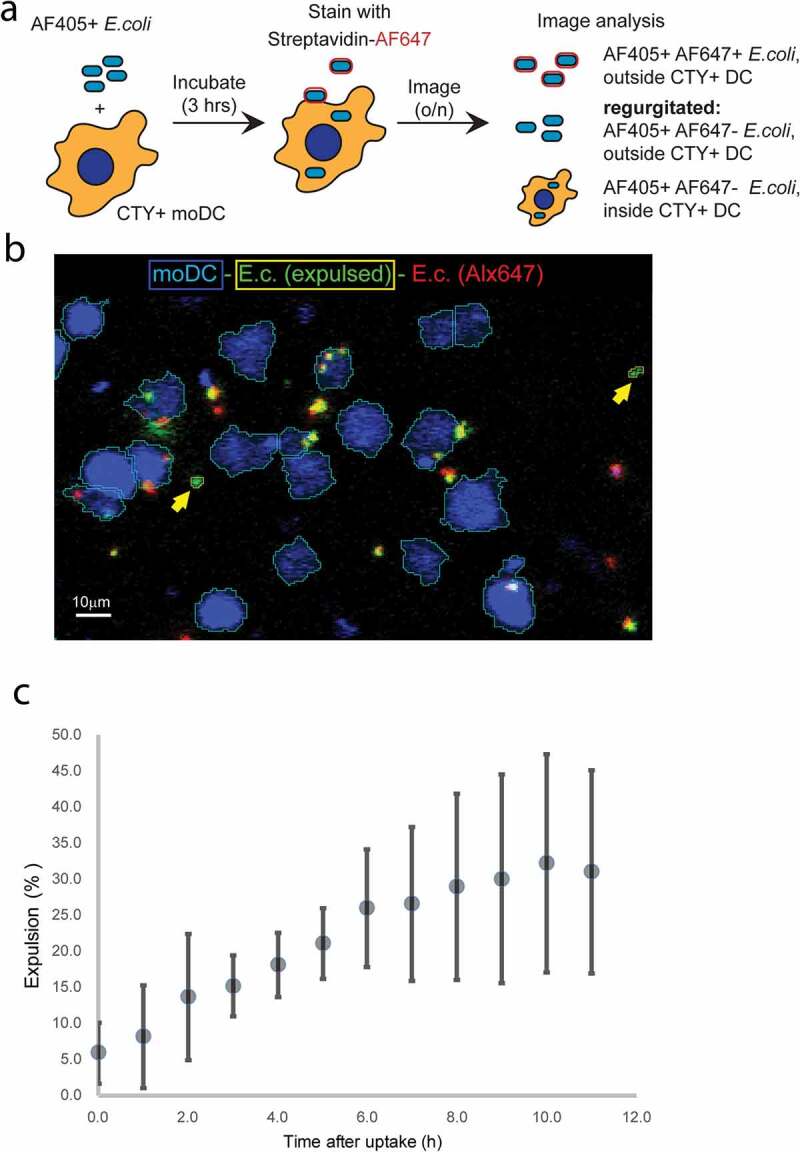
Confocal microscopy-based dynamic analysis of *E. coli* expulsion.

To quantify *E. coli* expulsion we resorted to live fluorescence microscopy (Figure 6). Cell Trace Yellow (CTY)-labelled moDC were pulsed for 3 hours with biotinylated AF405-labelled *E. coli*. Subsequently, the moDC were washed, after which remaining extracellular *E. coli* were labelled at 4°C with streptavidin-AF647. The moDC were then chased and continuously monitored for 11 hours by live fluorescence microscopy (Figure 6(a)). The AF405 signal was used to segment all *E. coli* objects (AF405^+^), and CTY fluorescence was used to segment moDC occupied areas in the images (CTY^+^). AF405-positive *E.coli* that were also labelled with AF647 (AF405^+^AF647^+^) were classified not to be phagocytosed directly after pulse loading. Phagocytosed *E. coli* were classified as AF405^+^CTY^+^AF647^−^ objects. Automated logical gating with different colour-combinations was used to identify and follow individual events during the chase (Figure 6(b)). Phagocytosed *E. coli* were identified to be expulsed when appearing outside CTY-labelled moDC during the 11-hour chase as AF405^+^CTY^−^AF647^−^ objects. The percentage of phagocytosed *E. coli* that was expulsed increased over time, reaching 32.2±15.1% (mean ± SEM from 3 independent experiments) after 11 hours (Figure 6(c)).

(a) Schematic presentation of the experiment. CTY-labelled moDC were incubated for 3hours with *E. coli* that were labelled with both biotin and AF405. Subsequently, moDC were pelleted, labelled on ice with streptavidin-AF647, and washed. The cells were then transferred to a confocal microscope and followed up to 11hours in a 5% CO_2_ containing atmosphere at 37°C by automated live confocal fluorescence microscopy imaging at 2min intervals. CTY, AF405 and AF647 labelled objects were isolated by image segmentation and defined *E. coli* inside moDC (AF405^+^CTY^+^AF647^−^), *E. coli* located outside moDC after 3 hour pulse loading (AF405^+^CTY^−^AF647^+^), and *E. coli* that were expulsed during the 11 hour chase (AF405^+^CTY^−^AF647^−^). (b) Representative still image after 10-hour chase from one out of three independent experiments. MoDC are depicted in blue (outline cyan), cell boundaries were automatically drawn. Phagocytosed *E. coli* are green in a blue background, expulsed green *E. coli* are outside blue areas, outlined in yellow, and indicated by yellow arrows. *E. coli* that were not phagocytosed after pulse loading of the moDC are stained both red and green. *E. coli* that was not phagocytosed after pulse loading but taken up during the chase is represented by the red dots in a blue background. (c) Kinetics of *E. coli* expulsion. Expulsed *E. coli* (AF405^+^, CTY^−^, AF647^−^) was determined as a percentage relative to all AF405^+^, AF647^−^*E. coli* (within and outside CTY background). The plot represents data from three independent experiments (mean ± SEM).

Importantly, the rate of expulsion of phagocytosed *E. coli* correlated with the *E. coli* stimulated release of EV associated HLA-I, HLA-II, CD81 and CD63, further supporting the idea that exosomes are expulsed by *E. coli* containing phagosomes that fuse with the plasma membrane.

## Discussion

We here demonstrate that up to ~30% of *E. coli* that were phagocytosed by moDC were expulsed back into the culture medium (Figure 6), and that phagocytosis by these cells stimulated the release of EV associated markers up to sixfold with comparable kinetics (Figure 4). The concomitant release of phagocytosed *E. coli* and small EV, together with the accumulation of HLA II and CD63, well-established exosomal markers, in discrete puncta within the perimeter of *E. coli* containing phagosomes (Figure 2), and the association of HLA II and CD63 positive puncta on regurgitated bacteria (Figure 3), strongly suggests that the released EV were generated as ILV by inward budding into phagosomes, and secreted as exosomes as a consequence of fusion of phagosomes with the plasma membrane. In contrast to CD63 and CD81, the tetraspanin CD9 is thought to be primarily associated with plasma membrane-derived microvesicles (MV) rather than with exosomes [12], and the release of CD9 was indeed not stimulated by *E. coli* (Figure 4), consistent with enhanced release of exosomes rather than MV. We were unable to directly visualize secretion of exosomes from phagosomes, as the required live cell technologies are not advanced enough yet. We attempted but failed to detect phagosome-plasma membrane fusion using tetraspanin-based pH-sensitive optical reporters in combination with live total internal reflection fluorescence microscopy, a technique that was recently developed by Verweij and co-workers to demonstrate exosome secretion by MVB [32]. This technique relies on sensing a shift in pH of the environment by the pH sensitive fluorescent probe when expelled from acidic endocytic compartments into a pH neutral extracellular environment. However, acidification of phagosomes in DC is limited by NOX2 [33], and TLR4 engagement restrains phagosome fusion with lysosomes to prevent full antigen degradation and promote cross-presentation [34]. This method thus cannot be applied to monitor the fusion of pH neutral phagosomes with the plasma membrane. Consistent with our observations for moDC, DC protruding into the gastrointestinal epithelium has been shown to secrete apically phagocytosed *E. coli* at their basolateral side [35]. The concept of non-lytic extrusion of phagosomal content has also been demonstrated for macrophages after phagocytic uptake of Cryptococcus neoformans [21,24], Candida albicans [23], or yeast [22]. Similar to phagosomes, also autophagosomes can secrete their contents. For example, uropathogenic *E. coli* was demonstrated to be expelled from infected bladder epithelial cells through expulsion of the content of autophagosomes [25]. In the same study, transmission electron microscopic pictures revealed the presence of small vesicles within the autophagosomal lumen, between its delimiting membrane and the autophagocytosed pathogen, consistent with a pre-exosomal identity [25]. Similar to phagosomes in DC, these autophagosomes have a neutral pH, which prevents degradation of their content by lysosomal proteases and stimulates exocytosis via TRP channel 3 (TRPML3), a transient receptor potential cation channel, resulting in expulsion of the bacteria together with associated exosomes [25]. Triggering of TLR4 in these autophagosomes stimulates polyubiquitination of TRAF, which on its turn stimulated RalGDS, a guanine nucleotide exchange factor (GEF), to assemble the exocyst complex [36]. Whether similar mechanisms drive the extrusion of phagosomes by DC or other cell types remains to be established. However, the release of exosomes by gastroendothelial cells was stimulated by infection with the protozoan parasite Cryptosporidium parvum, in a process involving TLR4/IKK2 signalling and a SNAP23-dependent exocytosis [18]. Interestingly, the secretion of exosomes by MVB also relies on the plasma membrane SNARE SNAP23 [32], suggesting parallel mechanisms of exosome secretion by MVB and phagosomes.

DC-derived EV are capable of inducing T-cell responses, through presentation of antigenic peptides on MHC molecules [2,4,5,10,11,17,37,38]. We recently demonstrated that non-cognate interactions between DC and bystander T-cells modulates third party antigen-specific T-cell responses via EV, possibly also involving EV mediated transfer of miR-155 [17]. The microRNA miR-155 is a critical regulator of adaptive immune responses [39], exemplifying a mechanism of how antigen presentation via intercellular transfer of EV may promote adaptive immunity.

Our current finding that exosomes can be generated in and secreted by phagosomes adds a new perspective of how exosomes may contribute in antigen presentation. HLA II molecules in pathogen loaded phagosomes can be expected to be preferentially loaded with pathogen-derived peptides, as compared to HLA II molecules residing in the hundreds or even thousands of other compartments composing the endocytic pathway within a single cell. As a consequence, HLA II molecules that are on exosomes that are generated as ILV in phagosomes can be expected to be preferentially loaded with pathogen derived peptides. In contrast, HLA II molecules that are on the plasma membrane are preferentially loaded with self-peptides as they are mostly recruited from all other, pathogen lacking, endocytic compartments. Hypothetically, the peptidome that is presented by HLA II molecules from isolated exosomes versus plasma membrane could be determined by mass spectrometry [40,41]. However, this is technically challenging, if not impossible at this time, given the limitations for obtaining sufficient quantities of exosomes from human moDC as source for peptide HLA II complexes. Moreover, separation of exosomes from plasma membranes is complicated by the fact that many EV are associated with the plasma membrane of their originating cell, either by tetherin [42] or after being recruited via integrin binding [43]. Further optimization of the separation of exosomes and plasma membranes, as well as of the isolation of peptide-MHC complexes from exosomes and the sensitivity of MS techniques, may enable such experiments in the future.

In conclusion, we hypothesize that HLA II molecules on exosomes that are secreted by phagosomes are preferentially loaded with pathogen-derived peptides, and thus excellently suited to stimulate adaptive immune responses. Importantly, T-cell receptors undergo dimerization before activation and this property might be essential for T-cell activation [44]. Consistent with this idea, it has been proposed that dimerization of MHC II molecules might be critical for TCR dimerization and T-cell activation [45,46]. However, given that less than 0.1% of all MHC II molecules on antigen loaded DC are loaded with a TCR-specific peptide [47], the probability that two identical peptide–MHC complexes reside in the same microdomain at the plasma membrane is neglectable. Preferential loading of pathogen derived peptides onto exosome associated MHC II in phagosomes may solve that problem, as this would both increase the density and force proximity of such pathogen peptide-MHC II complexes. This scenario could apply to DC exosomes that after secretion remain associated to the plasma membrane of their producing cell as well as to secreted exosomes that are recruited by bystander antigen presenting cells, resulting in rapid dissemination of the immune response.

## Supplementary Material

Supplemental Material
