# Supplementary figures and images for: Dendritic cells release exosomes together with phagocytosed pathogen; potential implications for the role of exosomes in antigen presentation
Source: J Extracell Vesicles. 2020 Jul 26;9(1):1798606. doi: 10.1080/20013078.2020.1798606 (PMC7480536; doi:10.1080/20013078.2020.1798606)

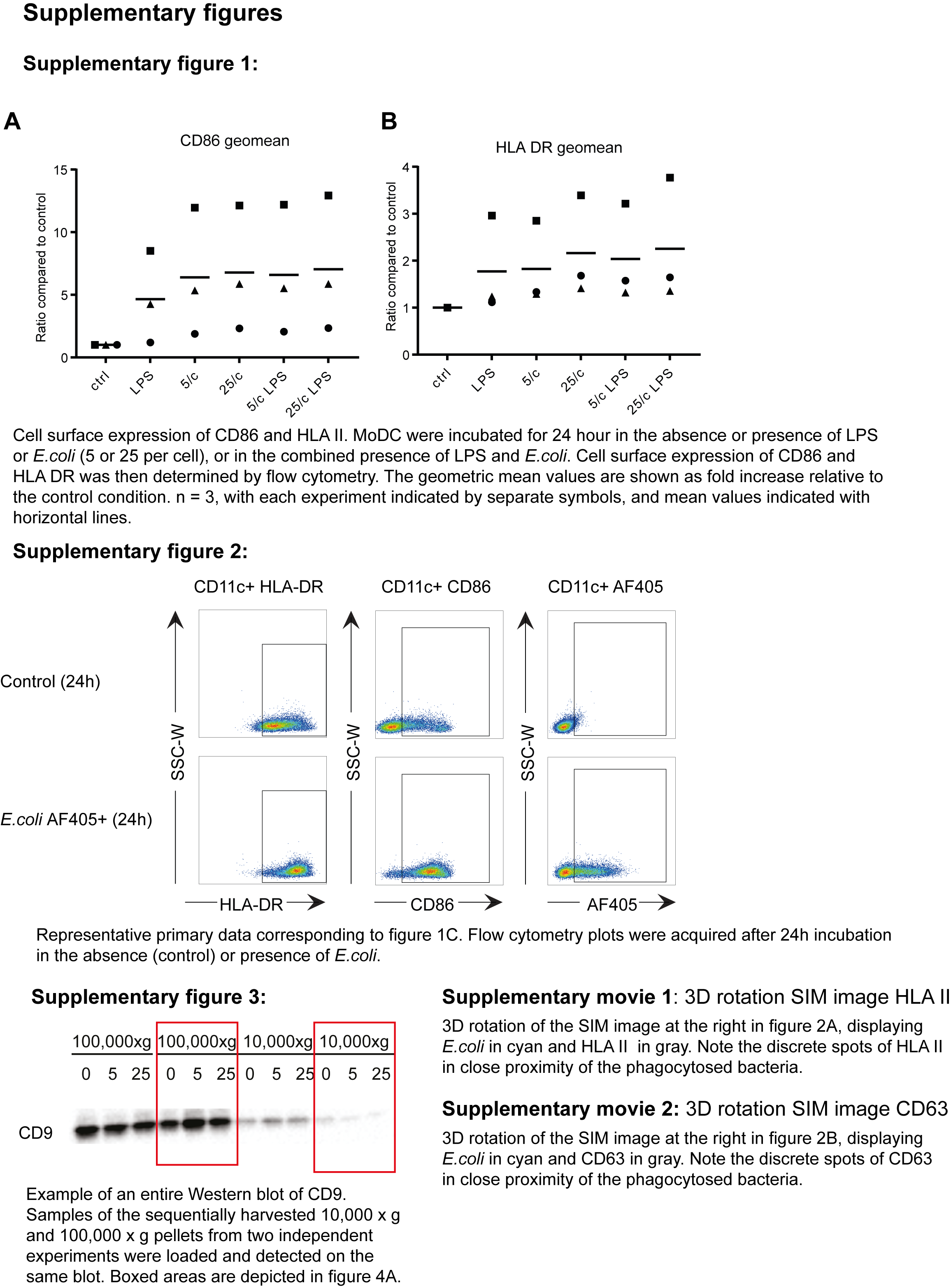

Supplement: Supplemental Material [file ZJEV_A_1798606_SM0582.zip › supplementary/20200611 Supplementary figures.tif]
